# Targeting Resident Memory T Cells for Cancer Immunotherapy

**DOI:** 10.3389/fimmu.2018.01722

**Published:** 2018-07-27

**Authors:** Charlotte Blanc, Sophie Hans, Thi Tran, Clemence Granier, Antonin Saldman, Marie Anson, Stephane Oudard, Eric Tartour

**Affiliations:** ^1^INSERM U970, Paris Cardiovascular Research Center (PARCC), Université Paris Descartes, Paris, France; ^2^Hôpital Européen Georges Pompidou, Department of Medical Oncology, Assistance Publique des Hôpitaux de Paris, Paris, France; ^3^Hôpital Européen Georges Pompidou, Laboratory of Immunology, Assistance Publique des Hôpitaux de Paris, Paris, France

**Keywords:** resident memory T cells, cancer vaccine, immune checkpoint molecule, mucosal route of vaccination, immunotherapy

## Abstract

A novel population of memory CD8^+^ T cells called resident memory T cells (T_RM_) has been identified based on their phenotype (CD103, CD69) and on their local tissue residency without recirculating in the blood. These cells have been implicated in protective immune response against pathogens in both animal models and humans. Their role in cancer is just emerging as a key player in tumor immunosurveillance. Many properties of these cells suggest that they could control tumor growth: (i) they respond much faster to reexposure to cognate antigen than circulating memory cells, (ii) they express high levels of cytotoxic molecules, and (iii) they are enriched in tumor-specific T cells in close contact with tumor cells. T_RM_ are present in many human cancers and are associated with a good clinical outcome independently of the infiltration of CD8^+^ T cells. It has been recently shown that the efficacy of cancer vaccines depends on their ability to elicit T_RM_. In adoptive cell therapy, the transfer of cells with the ability to establish T_RM_ at the tumor site correlates with the potency of this approach. Interestingly, T_RM_ express immune checkpoint molecules and preliminary data showed that they could expand early during anti-PD-1 treatment, and thus be considered as a surrogate marker of response to immunotherapy. Some cues to better expand these cells *in vivo* and improve the success of cancer immunotherapy include using mucosal routes of immunization, targeting subpopulations of dendritic cells as well as local signal at the mucosal site to recruit them in mucosal tissue.

## Introduction

After studying herpes simplex virus infection and lymphocytic choriomeningitis virus infection, two groups reported that memory T cells remain in dorsal root ganglia and small intestines, respectively, without recirculating through the blood (1, 2). These cells were called tissue-resident memory T cells (T_RM_). These T_RM_ cells may persist for a long time and represent one of the main lymphocyte populations in adults (3, 4).

T_RM_ cells originate from a common KLRG^neg^ memory precursor cell that also gives rise to circulating central and effector memory CD8 T cell populations (5). These cells share TCR repertoires (6).

T_RM_ cells from different tissues were transcriptionally related (5) with a core marker (CD69, CD103, and CD49a) both in mice and humans. However, subpopulations of T_RM_ differing by the expression of these markers and exhibiting additional markers also exist. For example, CD49a is expressed by only 15% of T cells from the human skin. The chemokine receptor CCR8 and the CD8αα homodimer are expressed only in skin T_RM_ cells, while the aryl hydrocarbon receptor (AHR) is expressed in gut and skin T_RM_, but not in lung T_RM_ (5, 7). This phenotypic heterogeneity extends to functional heterogeneity even within a same organ. For example, it has been shown that the airway T_RM_ has a poor *in vivo* proliferative and cytolytic ability, when they were compared with lung T_RM_, while IFNγ are produced faster by CD8 T_RM_ compared to systemic effector CD8^+^ T cells (8). In addition, T_RM_ in the airway has a short half-life (less than 1 month) whereas T_RM_ in lung parenchyma may persist for several months or years (9).

T_RM_ cells express high levels of protein associated with tissue retention, such as RGS-1 and RGS-2, both known as G protein-coupled inhibitors. By contrast, they display low levels of sphingosine-1-phosphate receptor 1 (S1PR1) and CCR7 (5, 10), which are indispensable for tissue exit. Various molecules expressed by T_RM_ may explain their long survival in tissue. Indeed, anti-apoptotic factors such as Bcl-2 could be detected in T_RM_ (5). In the presence of exogenous free fatty acids (FFAs), CD8^+^ T_RM_ cells exhibited high levels of mitochondrial oxidative metabolism. This feature was not observed in central memory CD8^+^ T cells. *Fabp4* and *Fabp5* (*Fabp4*/*Fabp5*) proteins favor FFA uptake by CD8^+^ T_RM_ cells. Their specific deficiency on T cells decreased the survival of T_RM_ *in vivo* (11).

Downregulation of T-bet, likely induced by TGF-β and T-box proteins Eomesodermin, is required for T_RM_ differentiation, but residual levels of T-bet for maintaining IL-15R are crucial for long-term T_RM_ function and survival in the skin, kidney, and salivary gland (12). However, IL-15 is not required for their maintenance in the small intestine or female reproductive tract (FRT) (5).

Aryl hydrocarbon receptor and Notch activity are also required for the maintenance of CD103^+^ T_RM_ cells (13, 14). Recent studies by Milner et al. identified the transcription factor Runx3 as a master regulator for inducing and maintaining CD8^+^ T_RM_ by reducing T_RM_ apoptosis (15).

In addition, in some tissue localizations (e.g., brain or lung), the presence of antigen is required for T_RM_ establishment (16, 17). By contrast, local inflammatory signal without antigenic stimulation may favor systemic CD8^+^ T cells to adopt T_RM_-like characteristics in skin, nasal tissue, and FRT (18).

T_RM_ have all the features of memory CD8^+^ T cells (CD45RA^−^CD62L^−^CD28^−^CD27^−^CCR7^−^) (19, 20). It has been clearly established that, at least in some tissues, T_RM_ cells might persist without the secondary recruitment of systemic effector memory T cells (21).

## Properties of T_RM_ that May Explain their Role in a Tumor Context

Various studies have shown that T_RM_ cells respond much faster to reexposure to cognate antigen than circulating memory cells [either TEM (effector memory T cells) or TCM (central memory T cells)] (22, 23). In addition, T_RM_ underwent *in situ* division after local antigen challenge, triggered the recruitment of innate immune cells and recirculating memory T cells and thus regulated local immunosurveillance (22–24).

T_RM_ cells in non-small cell lung cancer (NSCLC) are preloaded with preformed mRNA encoding inflammatory cytokines (granzyme B, IFN-γ, and TNF) and with cytotoxic molecules (13). In ovarian cancer, CD103^+^ tumor-infiltrating lymphocytes (TILs) uniformly express TIA-1, a marker of potential cytotoxicity (25). In liver cancer, T_RM_ express high levels of perforin (26). CD49a expression has been demonstrated to characterize T_RM_ cells poised with cytotoxic function in the human epidermis (27).

In some tissues such as the brain or the lung, local antigen presentation is required to drive T_RM_ cell formation (17). In addition, CD103^+^ TILs express high levels of PD-1 (25), which has been reported to be a marker of antitumor TILs in melanoma (28). Indeed, after their sorting based on their expression of PD-1, CD8^+^ T cells that expressed this inhibitory receptor in melanoma patients identified those that preferentially recognized tumor cells (28, 29). From these results, it thus appears that in many localizations, T_RM_ may represent antitumor-specific T cells.

In healthy tissues such as the lungs, the skin, the reproductive tract, and the gut, T_RM_ cells localize within the epithelial layer. CD103^+^ TILs were preferentially localized in epithelial regions of tumors in close contact with tumor cells, likely due to the natural interaction between CD103, and its ligand, E-cadherin, expressed by tumor cells, may explain that CD103^+^ TIL were rather found in close contact with the tumor cells rather than in the stroma (25, 30).

Finally, it has been shown that T_RM_ represent an effective *in situ* first line of defense to tissue-specific infections and are implicated in protective immune responses against many pathogens in both animal models and humans. It is thus tempting to extrapolate their role from infectious models to cancer (31).

## T_RM_ in the Natural Course of Tumor

T_RM_ are present in many human cancers (NSCLC, ovarian cancer, bladder cancer, endometrial cancer, melanoma, etc.).

Overall, they are associated with a good clinical outcome (19, 32). Interestingly, the impact of T_RM_ on survival was independent of the infiltration of CD8^+^ T cells. Indeed, we have shown in a multivariate analysis (33) that intratumoral CD103^+^CD8^+^ T cells correlate with a better survival in NSCLC patient (33). Confirming our results, a greater number of intratumoral T_RM_ cells correlated with a better survival in lung cancer, cervical cancer, and melanoma, independently of that conferred by total CD8^+^ T cells (34–36).

Finally, intratumoral CD8^+^ T cells not expressing CD103 were associated with poor prognosis, as observed in tumors not infiltrated by CD8^+^ T cells (25).

The localization of T_RM_ inside the tumor may be a parameter to take into account to assess their impact on the control of the tumor. Indeed, intraepithelial CD103 but not intra-stromal CD103 correlated with better overall survival and absence of relapse in a basal-like subtype of breast cancers (30). In many of these studies, the CD103 marker was analyzed and not really T_RM_ (CD103^+^CD8^+^ T cells). Since CD103 is also expressed by CD4^+^ T cells, innate lymphoid cells, NK cells, and dendritic cells (DCs), it could introduce a bias in the interpretation of the results.

Interestingly, the genetic variability of TCRs from resident memory T cells between different metastatic lesions from the same patient was greater than the variance in mutational or neoepitope load in tumor cells (37). This absence of equilibration between tissue-resident TCR within individual metastases may affect the clinical results of immunotherapy at the various sites and explain mixed clinical response.

## Role of T_RM_ in the Efficacy of Cancer Vaccine

Using a model of orthotopic head and neck or lung cancer, we showed that only the intranasal route of immunization elicited local T_RM_. By means of parabiosis experiment or the use of the FTY720 inhibitor, which downregulates the S1PR1 receptor and blocks the recruitment of circulating memory T cells in the tissue, we demonstrated that the T_RM_ alone could partially control the growth of the tumor (33, 38). It was also reported that an intravaginal boost with an HPV vaccine after a systemic (intramuscular) prime was more efficient at eliciting local cervical T_RM_ cells, which led to a better overall mouse survival after a tumor challenge than that observed with an intramuscular boost (39).

In melanoma patients vaccinated with a mixture of Melan-A peptide combined with Montanide and CpG, the ability to elicit anti-Melan A CD8^+^ T cells expressing VLA-1, a surrogate marker of T_RM_, was correlated with better survival (40).

Treatment of breast DCs with β-glucan—a ligand of dectin-1 reprogrammed DC with an upregulation of ITGB8, an integrin which binds the latent domain (LAP) of TGF-β, and which after its cleavage constitutes the main mechanism of TGF-β activation *in vivo*. Administration of DC treated with β-glucan curdlan or its direct intratumoral delivery induced intratumoral antitumor CD8^+^ T cells expressing CD103, which inhibit tumor progression in a humanized mouse model of breast cancer (41).

While these examples strongly suggest the role of T_RM_ in the protection generated by cancer vaccine, it has to be kept in mind that FTY720 experiments showed that the recruitment of circulating effector memory T cells increased the efficacy of T_RM_ after mucosal vaccine (33). Conversely, Dr. Sancho’s group reported that, while both T_RM_ cells and circulating memory T cells play a role in tumor immunosurveillance, the presence of T_RM_ cells improves vaccine efficacy (42).

## Role of T_RM_ in Adoptive T Cell Therapy

Mucosal CD103^+^CD8^+^ T cells elicited by reprogrammed DC with β-glucan curdlan can reject an established tumor and this effect is inhibited by the blockade of CD103 (41).

The establishment of T_RM_ cell populations in various normal tissues and in cancer required the expression of Runx3 (15). In a preclinical model of melanoma, CD8^+^ TIL not expressing Runx-3 did not accumulate in tumor microenvironment, resulting in uncontrolled tumor growth and low survival. By contrast, when antitumor CD8^+^ T cells that overexpress Runx3 were transferred *in vivo*, tumor growth was inhibited, and mice survival improved (15). Thus, the adoptive T cell therapy of T_RM_ seems a promising strategy.

## Role of T_RM_ in Cancer Immunotherapy Based on the Blockade of Immune Checkpoint Molecules

T_RM_ from healthy organs (brain, gut, lung, and skin) or localized in tumors (NSCLC, melanoma, etc.) express higher amounts of inhibitory receptors (PD-1, Tim-3, CTLA-4, NKG2A, BTLA, LAG-3, SPRY1, adenosine receptor A2AR, CD39, CD101, and 2B4) and costimulatory molecules (CD27, ICOS, SIRPG, and CD137) than peripheral memory CD8^+^ T cells or CD8^+^CD103^neg^ TIL (5, 13, 20, 34, 37).

However, depending on the tumor localization, the profile of immune checkpoint molecules detected on T_RM_ may vary. For example, T_RM_ derived from NSCLC and melanoma did not express membrane CTLA-4 (19, 35), and in ovarian cancer, PD-1^+^CD103^+^CD8^+^ T cells exhibited a weak expression of other exhaustion-associated markers, such as CTLA-4, LAG-3, and TIM-3 (32).

TCGA analysis of cervical cancer data shows that *CD103* (*ITGAE*) expression correlates with the usual T cell genes such as *CD8A*, but more interestingly also with T cell activation and exhaustion markers such as CTLA-4, CD137, PD-1, and PD-L1 (36).

Transcriptomic analysis of T_RM_ also reported the expression of genes with well-recognized inhibitory functions in T cells, such as the dual specificity phosphatase DUSP6, which turns off MAP kinase signaling, as well as IL-10 (20). However, despite high expression of checkpoint inhibitors, several arguments show that T_RM_ cells from infected organs or tumors are not terminally exhausted. Indeed, T_RM_ in the hepatitis B virus-infected human liver co-express PD-1 and CD39 at high levels, but they readily produce IFN-γ, TNFα, and IL-2 after *in vitro* stimulation (26).

In addition, when T_RM_ cells sorted from lung carcinomas were co-cultured with autologous tumor cells, their cytotoxic activity was enhanced in the presence of anti-PD-1 mAb (19).

In a preclinical model, administration of anti-PD-1 antibody concomitantly with Tcm transfer (which converts to T_RM_) in a tumor therapy setting inhibited the growth of s.c. MC38-OVA tumor and i.d. B16-OVA tumor when compared with the adoptive T cell therapy with Tcm cells only. Interestingly, after anti-PD-1 therapy, the number and frequency of TIL with a T_RM_ phenotype were increased more than 10-fold within the CD45^+^ cells in both tumor settings (42).

In humans, tumor-resident CD8^+^ T cells significantly expanded early during anti-PD-1 treatment (35). There was a significant difference in their numbers (T_RM_) early during treatment between those who responded to the treatment and those who did not respond (35). In line with these results, Wei et al. showed that T cell clones that expanded during anti-PD-1 treatment expressed high levels of CD69, PD-1, LAG-3, and CD45RO, an identical phenotype to the tumor-resident CD8^+^ T cell population (43).

## Cues to Elicit T_RM_ to Improve Cancer Immunotherapy

**Figure 1:**
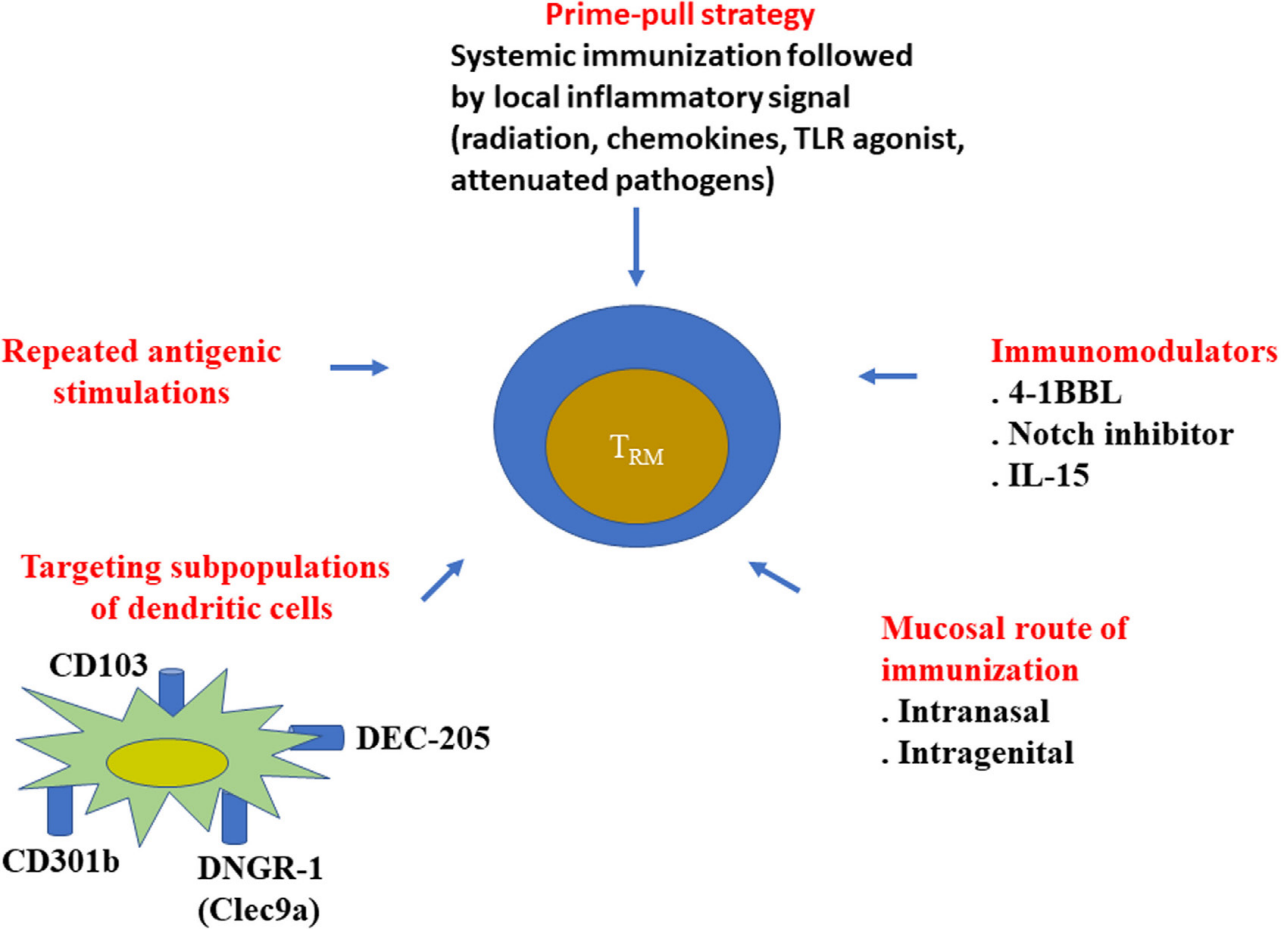
Various strategies to elicit resident memory T cells. Various approaches have been proposed to better elicit resident memory CD8^+^ T cells, especially during vaccine administration. It has been well demonstrated that mucosal routes of immunization (intranasal, intragenital, etc.) better induce local CD8^+^ T_RM_, than the conventional systemic route (intramuscular, subcutaneous, etc.). Local inflammatory signal after a systemic vaccination (prime-pool protocol) could also recruit CD8^+^ T cells in the tissue. However, the phenotype of the recruited cells was not always checked to determine if they represented *bona fide* T_RM_.

From these results, it is clear that T_RM_ are involved in the efficacy of different cancer immunotherapy strategies. A field of future investigation will rely on the development of new strategies to induce and amplify T_RM_ (Figure 1).

### Route of Immunization

Compelling experiments demonstrate the crucial role of the route of vaccination to elicit tissue-resident memory T cells both during natural infection and after vaccine administration.

Indeed, various vaccine studies showed that intravaginal immunization or a systemic prime followed by a mucosal vaginal boost maximized the induction of genital T_RM_ (31). Intranasal vaccination with a recombinant cytomegalovirus vector encoding the respiratory syncytial virus (RSV) matrix (M) or with BCG protein also generated robust and durable tissue-resident effectors that were undetectable after intraperitoneal or subcutaneous vaccination (44, 45).

### Local Signal to Favor the Recruitment of T_RM_

In mice, cancer vaccine synergizes with local radiation to favor the recruitment of intratumoral antitumor CD8^+^ T cells, some of them exhibiting a T_RM_ phenotype (36, 46).

Local injection of Toll-like receptor agonists or of selected chemokines *via* the modification of the expression of selectins, integrins, and chemokines could also enhance the recruitment of CD8^+^ T cells in the tissue and at local tumor site. This concept has been assessed *in vivo* by the “prime and pull” strategy, which comprises two steps: conventional systemic immunization to induce T-cell responses in the blood (prime), followed by secondary recruitment of effector T cells by means of local chemokine injection into the mucosal genital tract (pull). This prime-pull strategy succeeded in establishing a long-term residency and thus favored protective immunity. In mice, this prime and pull strategy was shown to decrease the diffusion of infectious herpes simplex virus 2 (HSV-2) into the sensory neurons and to be efficient to control clinical disease (18). In line with these results, after systemic administration of a vaccine, an intravesical administration of Ty21, a live bacterium used against typhoid fever or an intravaginal administration of CpG resulted in the accumulation of local specific CD8^+^ T cells and led to tumor regression (47, 48).

This prime-pull strategy is thus an attractive strategy, but the phenotype of these intratissular-recruited CD8^+^ T cells has not been fully established. In addition, it has not been reported whether these cells represent *bona fide* T_RM_.

### Targeting DCs to Elicit T_RM_

Optimal generation of T_RM_ cells requires CD103^+^ DCs in non-lymphoid tissues, which are dependent on the transcription factor BATF3 for their development, as well as mouse CD8α^+^ DCs in lymphoid organs (49). CLEC9A (DNGR-1) and DEC-205 are highly expressed by CD103^+^ DC and CD8αDC. Intranasal delivery of targeting antibodies (DEC-205 or CLEC9A) proved highly protective against lethal influenza challenge (50). This protection is based on both the initiation of T-cell priming in the lung and the enhancement of local presentation and differentiation of T_RM_ cell (50).

CD301b^+^ DCs also promote CD8^+^ T cells with a T_RM_ phenotype which control genital HSV-2 infection (51).

In humans, lung-resident CD1c^+^ DCs drove CD103 expression on effector CD8^+^ T cells by displaying membrane-bound TGF-β1 (52).

### Immunomodulators

Intranasal delivery of 4-1BBL in combination with an adenovirus encoding an influenza nucleoprotein to naïve mice elicits systemic effector memory CD8^+^ T-cell expressing IL-7Rα, as well as an intraparenchymal lung CD69^+^CD8 T_RM_ population, which comprised both CD103^+^ and CD103^neg^ cells. Moreover, physiologically, during primary influenza infection, T cells deficient for 4-1BB do not differentiate into lung-resident T_RM_ population (53).

Formalin inactivated RSV plus CpG plus L685,458, an inhibitor of Notch signaling, promoted protective CD8^+^ lung tissue-resident memory T cells (54).

IL-15 complexes delivered locally to mucosal tissues without reinfection are an effective strategy to enhance establishment of tissue-resident memory CD8 T cells within mucosal tissues (55).

Our group showed that cancer vaccine administered by the intranasal route in combination with an anti-TGFβ decreased the number of T_RM_ without having any impact on T effector cells, and partially inhibited the protective effect of the vaccination (33).

### Repeated Antigenic Stimulation

We and other showed that the density of T_RM_ in tissues and tumors progressively increased after each immunization (33). Prime boost immunization with recombinant adenovirus expressing HPV16 E7 protein *via* a homologous (intravaginal) or heterologous (intramuscular followed by intravaginal) route of immunization elicited more T_RM_ in the cervicovaginal mucosa than did a single priming by the intravaginal route (56). Multiple infections also result in more widespread or global T_RM_ dissemination (21).

## Conclusion

In the recent past, T_RM_ have been emerging as having an important role in cancer immunotherapy based on cancer vaccine, adoptive cell therapy, and the blocking of the interaction of immune checkpoint molecules with their ligands. In the next few years, it will be necessary to better distinguish subpopulations of T_RM_ in different tissues with different phenotypes and functions. The vast majority of studies focus(ed) on CD8^+^ T_RM_. Further analysis of CD4^+^ T_RM_ with phenotype and function that may be different from CD8^+^ T_RM_ should be performed. Optimization of immunotherapy strategies to induce these T_RM_ is already the subject of ongoing work. Their role as a biomarker of responses to immunotherapy is also being evaluated based on preliminary encouraging results.

## Author Contributions

All authors listed have made substantial and direct intellectual contributions to the work and approved it for publication.

## Conflict of Interest Statement

The authors declare that the research was conducted in the absence of any commercial or financial relationships that could be construed as a potential conflict of interest.
